# CRISPR/Cas9-based liver-derived reporter cells for screening of mPGES-1 inhibitors

**DOI:** 10.1080/14756366.2019.1587416

**Published:** 2019-03-18

**Authors:** Zhanfei Chen, Xiaoling Cai, Man Li, LinLin Yan, Luxi Wu, Xiaoqian Wang, Nanhong Tang

**Affiliations:** a Fujian Institute of Hepatobiliary Surgery, Fujian Medical University Union Hospital, Fuzhou, China;; b Key Laboratory of Ministry of Education for Gastrointestinal Cancer, Research Center for Molecular Medicine, Fujian Medical University, Fuzhou, China

**Keywords:** CRISPR/Cas9, liver-derived cells, mPGES-1, inhibitor

## Abstract

mPGES-1 is a terminal rate-limiting enzyme responsible for inflammation-induced PGE2 production. The inhibition of mPGES-1 has been considered as a safe and effective target for the treatment of inflammation and cancer. However, a specific, efficient, and simple method for high-throughput screening of mPGES-1 inhibitors is still lacking. In this study, we developed a fluorescence imaging strategy to monitor the expression of mPGES-1 via CRISPR/Cas9 knock-in system. Immunofluorescence colocalisation, Sanger sequencing, RNAi, and IL-1β treatment all confirmed the successful construction of mPGES-1 reporter cells. The fluorescence signal intensity of the reporter cells treated with four conventional mPGES-1 inhibitors was considerably attenuated via flow cytometry and fluorescent microplate reader, demonstrating that the reporter cells can be used as an efficient and convenient means for screening and optimising mPGES-1 inhibitors. Moreover, it provides a new technical support for the development of targeted small molecule compounds for anti-inflammatory and tumour therapy.

## Introduction

Prostaglandin E (PGE2), a mediator of the body's inflammatory response, is closely related to many inflammation-related diseases, such as cardiovascular, joint, and neurodegenerative diseases, as well as cancer^1–3^. PGE2 is one of the main metabolites of arachidonic acid (AA), which is mainly involved in phospholipase A2, cyclooxygenase (COX-1 and COX-2), and PGE2 synthase (cPGES, mPGES-1, and mPGES-2)^4^. In non-inflammatory response, the body relies on COX-1 (non-inducible), cPGES, and mPGES-2 to produce basal levels of PGE2 to maintain organs’ physiological function. In chronic inflammation, AA produces an excess of prostaglandin H2 (PGH2) via COX-2 enzyme reaction. PGH2 is further converted into a large amount of PGE2 by terminal synthase mPGES-1^5–7^. Clinically, the use of COX-2 inhibitors can reduce the production of PGE2 and achieve anti-inflammatory effects. However, studies have shown that COX-2-specific inhibitors have many side effects (causing cardiovascular disease or gastrointestinal bleeding)^8^^,^^9^, which showcase the urgent need for the discovery of novel potent and safe anti-inflammatory drugs.

mPGES-1 can be inducible expression at the inflammation site, is also involved in the proliferation, invasion, and metastasis of cancer cells^1^^,^^7^^,^^10^. mPGES-1 inhibitors may present superior safety in comparison to existing anti-inflammatory drugs by preventing the synthesis of PGE2 without affecting the production of other prostaglandins. At present, a variety of structurally different inhibitors has been developed. For the selection of mPGES-1 inhibitors, enzyme-linked immunosorbent assay (ELISA) and high-throughput screening are common methods. The ELISA has high sensitivity and specificity and is often used to quantify PGE2 production. However, this method has many disadvantages, such as numerous factors affecting the results; the High-throughput screening requires complex instruments and reagents, which are costly. In recent years, CRISPR/Cas9 fixed-point editing gene technology has become popular worldwide, and its precise gene editing ability promotes the rapid development of genetic engineering^11–13^. Studies have shown that sgRNA-mediated cleavage of Cas9 protein and homologous recombination (HDR) pathway can insert tags into endogenous genes of cells to track the expression of such genes^14–16^. During HDR, the use of a homology arm length of 1 kb or longer can improve the targeting efficiency of Cas9 and reduce the risk of off target^17^. Therefore, our study used CRISPR/Cas9 technology to knock into the red fluorescent tdTomato gene and successfully constructed mPGES-1 fluorescent reporter cells. By fluorescently reporting cells, we visualised the expression and location of mPGES-1, which in turn provided an efficient and convenient means for screening and optimising mPGES-1 inhibitors. This method provided new technical support for the development of targeting small molecule compounds.

## Materials and methods

### Cell culture, transfection, and flow cytometry (FCM) analysis

Human hepatoblastoma cell line HepG2 (HB-8065, ATCC, Manassas, VA), HepG2.2.15 (donated by the laboratory of the First Affiliated Hospital of Fujian Medical University), hepatoma cell lines Huh7 (JCRB0403, Japan), HL7702 and BEL-7404 (Shanghai Institute of Cell Biology, Chinese Academy of Sciences), MHCC97H (Fudan University Hepatology Research Institute) were maintained in Dulbecco's modified Eagle medium (DMEM, Gibco, Carlsbad, CA) supplemented with 12% foetal bovine serum (FBS, Gibco, Carlsbad, CA).

The cells were seeded at a density of 5 × 10^5^ cells/well, and DNA transfection was performed in a six-well plate using Lipofectamine 2000 (Life Technologies, Carlsbad, CA) according to the manufacturer's instructions. The transfection ratio of the Donor vector PX459 (donated by the Key Laboratory of Ministry of Education for Gastrointestinal Cancer of Fujian Medical University): sgRNA vector was 3:1, and the co-transfected cells were screened for 2 week using 1 mg/mL G418. The red fluorescent PE intensity (the signal intensity of PE channel) of the reporter cells was detected using the BD Accuri C6 PlusTM platform. The materials used in the transfection experiments were interleukin-1β (IL-1β, ab9617, Abcam, Cambridge, UK) and small molecule inhibitors MK-886 and MF63 (Cayman Chemical, Ann Arbor, MI), 1,3-benzoxazol-5-amine and 2,5-dimethyl-celecoxib (Sigma-Aldrich, St. Louis, MO).

### Construction of PX459: sgRNA recombinant vector and donor recombinant vector

The Cas9 target site was identified using an online CRISPR design tool (crispr.mit.edu). Briefly, sgRNA was designed using the 250 bp DNA sequence of the human mPGES-1 gene flanking the stop codon (Table 1). In order to clone a single vector expressing PX459:sgRNA, the expression vector of PX459 expressing Cas9 and sgRNA was linearised by Bbs I digestion and gelatinised. A pair of annealed oligonucleotides (20 bp target sequence) were phosphorylated, annealed and ligated into linearised PX459. Finally, the vector is sequenced to ensure the correct sequence is obtained. The construction of the Donor plasmid was based on the plasmid pET32-2A-tdTomato-loxP-CAG-neo-loxP (donated by Dr. Wang Yunfang from the Stem Cell and Tissue Engineering Laboratory of Beijing Institute of Transfusion Medicine). The cell DNA was extracted and the target sequence was obtained by PCR using the upstream and downstream primers of the left arm and right arm (Table 1). The left arm target sequence was digested into pET32-2A-tdTomato-loxP-CAG-neo-loxP by endonuclease BglII and EcoRV and right arm sequences using endonucleases SacI and SalI. Finally, the vector is sequenced to ensure the correct sequence.

**Table 1.** List of oligonucleotides used in the present study.^a^

Oligonucleotides	Sequences (5′ → 3′)
sgRNA oligo sequence	
sgRNA1 F	CACCGGGTCTCCATGTCGTTCCGGT
sgRNA1 R	AAACACCGGAACGACATGGAGACCC
sgRNA2 F	CACCGAGAGCCGCCGTGGCTATACC
sgRNA2 R	AAACGGTATAGCCACGGCGGCTCTC
sgRNA3 F	CACCGGTCCTAACCCTTTTGTCGCC
sgRNA3 R	AAACGGCGACAAAAGGGTTAGGACC
sgRNA4 F	CACCGGGTATAGCCACGGCGGCTCT
sgRNA4 R	AAACAGAGCCGCCGTGGCTATACCC
sgRNA5 F	CACCGAGCCGCCGTGGCTATACCTG
sgRNA5 R	AAACCAGGTATAGCCACGGCGGCTC
sgRNA6 F	CACCGCCATGGGCCAAGAGCCGCCG
sgRNA6 R	AAACCGGCGGCTCTTGGCCCATGGC
Donor plasmid	
Left arm F	GA***AGATCT***CGAGGAGACGGTGTTATTGG
Left arm R	CG***GATATC***CAGGTGGCGGGCCGCTT
Right arm F	C***GAGCTC***CCAGCAGCTGATGCCTCCTTG
Right arm R	ACGC***GTCGAC***ACATCTCAGGTCACGGGTCT
Real-time RT-PCR	
mPGES-1 F	CCAGCCACTCAAAGGAACT
mPGES-1 R	ATCTCAGGTCACGGGTCTA
GAPDH F	AGGGCTGCTTTTAACTCTGGT
GAPDH R	TCTCGCTCCTGGAAGATGGTG
Cas9 F	CCGAAGAGGTCGTGAAGAAG
Cas9 R	GCCTTATCCAGTTCGCTCAG
T7E1 assay	
sgRNA targeting test F	ACTCTGGGGTATCCATCTCCTTGA
sgRNA targeting test R	CAGGAATCCAAGGGGCTAAGAAAC
Clone identification	
tdTomato F	CGTGAAGCACCCCGCCGACA
tdTomato R	CCGGTGCCATGCCCCAGGAA
Left arm-tdTomato F	TCCGTGACCTACACCCTG
Left arm-tdTomato R	GTAATCGGGGATGTCGG
RNAi	
PTGES-352	GGAUGCACUUCCUGGUCUUTT
	AAGACCAGGAAGUGCAUCCTT
PTGES-271	GGAACGACAUGGAGACCAUTT
	AUGGUCUCCAUGUCGUUCCTT

a Restriction sites are shown in bold and italics.

### RNA extraction and real-time RT-PCR analysis

Total RNA was extracted from the cells using TRIzol reagent (Thermo Scientific, Waltham, MA). The reverse transcription reaction was carried out by using a Transcriptor First Strand cDNA Synthesis Kit (Roche Diagnostics, Mannheim, Germany) with 3 μg of RNA at a final concentration of 20 μL. Quantitative real-time PCR was performed using an ABI 7500 real-time PCR system (Applied Biosystems, Foster City, CA) and a Fast Start Universal SYBR Green Master Mix (Roche, Mannheim, Germany). Glyceraldehyde-3-phosphate dehydrogenase (GAPDH) was used as an internal reference control. Primers for GAPDH, Cas9^18^ and mPGES-1 amplification are shown in Table 1, and each sample was analysed in triplicate. The relative expression levels of Cas9 and mPGES-1 were calculated by normalising endogenous GAPDH mRNA expression prior to comparative analysis using the 2^–ΔΔCt^ method as previously described^19^. All procedures follow the manufacturer's instructions.

### Western blotting

Thirty micrograms of protein was applied to 12% sodium dodecyl sulphate-polyacrylamide gel electrophoresis (SDS-PAGE) and electrophoretically transferred to a polyvinylidene fluoride (PVDF) membrane (Merck Millipore, Darmstadt, Germany). Western blots were incubated separately from a panel of specific antibodies including anti-mPGES-1 (ab62050, Abcam, Cambridge, UK) (1:3000 dilution) and anti-beta-actin (sc-47778, Santa Cruz Biotechnology, Santa Cruz, CA) antibody (1:2000 dilution). Immunoreactivity was detected using a chemiluminescent immunoblot immunoassay kit (Thermo Scientific, Waltham, MA) according to the manufacturer's instructions and recorded on a Hyperfine-ECL detection membrane. The amount of each protein was semi-quantitatively determined as the ratio of β-actin indicated on each gel.

### T7 endonuclease I (T7E1) assay

Cellular DNA was extracted 48 h after co-transfection of PX459:sgRNA2 and donor according to the T7E1 test kit instructions (GenePharma, Shanghai, China), using the T7E1 sgRNA targeting test F′ and R′ primers (Table 1). The PCR product (200 ng) was denatured (95 °C, 10 min) and then reannealed. Hybridised DNA-digested T7E1 (30 min at 37 °C) was analysed on an agarose gel.

### Immunofluorescence

For immunofluorescence staining, cells were first fixed with 4% paraformaldehyde, then permeabilised and blocked with blocking buffer (PBS containing 0.5% Triton X-100, 1% BSA, and 10% donkey serum). The cells were incubated with anti-mPGES-1 antibody (ab62050, Abcam, Cambridge, UK) overnight at 4 °C, and then incubated with fluorescent secondary anti-rabbit antibody (HS111, TransGen Biotech, Beijing, China) for 1 h at 37 °C in the dark. Cells were counterstained with 4′,6-diamidino-2-phenylindole (DAPI) and visualised using a Zeiss LSM 780 confocal microscope (Carl Zeiss Meditec AG, Jena, Germany). A suitable normal IgG antibody and a second antibody are provided as negative controls (NCs).

### Cell viability assay

Cell viability and detection of drug toxicity IC20 were assessed using the CCK-8 Assay Kit (Dojindo Laboratories, Kumamoto, Japan) and operated according to the instructions. WST-8 was measured spectrophotometrically at 450 nm and 600 nm (reference wavelength) using a SpectraMax M5 (Molecular Devices, Sunnyvale, CA). To eliminate the reference wavelength interference, “OD450 nm–OD600 nm” is the final value. The IC20 of MK-886, 1,3-benzoxazol-5-amine, 2,5-dimethyl-celecoxib, and MF63 was measured to be 9.75 μM, 169.22 μM, 31.21 μM, and 2.52 μM.

### RNA interference

All small interfering RNAs (siRNAs) of mPGES-1, including NC with no homology to known human genes, were chemically synthesised by GenePharma (Shanghai, China), and the sequences are shown in Table 1. One hundred picomoles of mPGES-1 siRNA or NC were used for transfection. Total RNA and protein were extracted 48 h after transfection according to the manufacturer's instructions.

### Fluorescence microplate reader assay

The cells were seeded at a density of 0.5 × 10^4^ per well in black 96-well plates with opaque walls and placed in a 37 °C incubator. After 24 h, the experimental group was treated with a small molecule inhibitor, and after 72 h, the fluorescence intensity of the cells was measured using a fluorescent plate reader (SpectraMax i3x). The excitation light wavelength was 554 nm, and the emission light wavelength was 581 nm.

### Detection of PGE2 secretion level

The level of PGE2 in the supernatant of the cells treated with inhibitors was detected by PGE2 ELISA kit (No. 514010, Cayman Chemical, Ann Arbor, MI), and the experimental operation was carried out according to the manufacturer’s instructions. The equation of the standard curve obtained in this experiment is “*y*=(*A* – *D*)/[1+(*x*/*C*)*^B^*]+*D*” (*A* = 111.74, *B* = 0.94, *C* = 35.99, *D*=–1.63, *R*^2^=0.99953). The ELISA data were obtained using SpectraMax M5 and the absorbance was detected at 412 nm.

### Statistical analysis

Analysis of variance (ANOVA) and *t*-test statistical analysis were performed using GraphPad Prism7 software (San Diego, CA). All values are expressed as mean ± standard deviation (SD) of replicates. A *p* value <.05 indicates statistical significance.

## Results

### Construction of mPGES-1 fluorescent reporter cells using CRISPR/Cas9 technology

**Figure 1.**
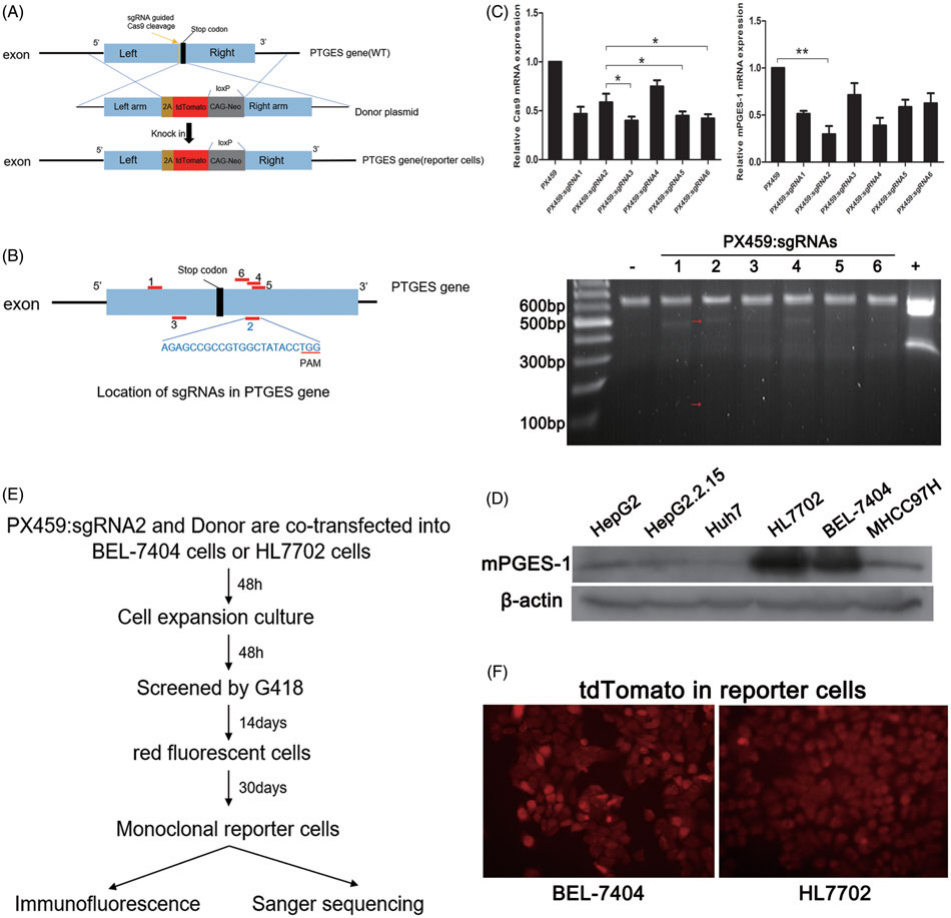
Construction of mPGES-1 fluorescent reporter cells using CRISPR/Cas9 technology. (A) CRISPR/Cas9 knock-in was used to construct mPGES-1 fluorescent reporter cells. 2A-tdTomato-loxp-CAG-Neo-loxp was integrated into the *PTGES* gene of chromosome to replace the stop codon to obtain the reporter cells stably expressing red fluorescence and G418 resistance. (B) Six sgRNAs were distributed in different positions of *PTGES* gene. (C) PX459: sgRNAs were transiently transfected into 293T cells, and DNA and RNA were extracted 48 h later. Three micrograms of RNA was reverse transcribed for real-time fluorescent quantitative PCR, and the group transfected with PX459 empty vector was used as a control. The value was set to 1, and *p< .05, **p< .005, *n* = 3. The extracted DNA was used for T7E1 assay, the red arrow was the strip after digestion, – was the negative control, and + was the positive control. (D) The basic expression of mPGES-1 protein in different hepatogenic cells. Thirty micrograms of total protein was used for Western blot, and β-actin was used as internal reference protein to normalise gray value. (E) The construction process of monoclonal fluorescent reporter cells. PX459: sgRNA2 and donor vector were co-transfected into cells, and screened by G418 for 2 weeks. Cells were picked in 96-well plates and expanded to obtain monoclonal cells stably expressing red fluorescence for subsequent validation. (F) Cells stably expressing red fluorescent protein were obtained by G418 screening.

To construct mPGES-1 reporter cells, we applied the principle of CRISPR/Cas9 knock-in gene editing (Figure 1(A)) to cotransfect mPGES-1 sgRNA recombinant vector with a homologous recombinant donor vector in liver-derived cells. A cell line stably expressing fluorescence was obtained via resistance screening. In the donor vector, the main functional sequence was left arm-(2A-tdTomato-loxp-CAG-Neo-loxp)-right arm. The left arm had a sequence of 1335 bp upstream of the stop codon. The right arm had a sequence of 1228 bp downstream of the stop codon. The sequence of 2A-tdTomato was the core part and replaced the stop codon. When the Cas9 protein functions, the sequence near the stop codon of the target gene mPGES-1 in the liver cancer cell breaks to form DSB. At this time, the left and right arms of the mPGES-1 stop codon in the donor vector integrate the core portion 2A-tdTomato (red fluorescent group) sequence into the genome of the cell by HDR. Then, the cells acquire neomycin resistance and stably express red fluorescent protein.

Six pairs of mPGES-1 sgRNAs were obtained by using the CRISPR design tool. The sgRNAs differed in position near the stop codon (Figure 1(B)) and were designated as sgRNA1 to sgRNA6 (Table 1). Six pairs of sgRNAs were constructed in PX459 to form six different sequences of Cas-sgRNA expression vector PX459, namely, PX459:sgRNA1∼PX459:sgRNA6. To test the editing efficiency of sgRNAs, PX459:sgRNAs were transiently transfected into the 293T cells, RNA and DNA were extracted 48 h after transfection. RNA was used to detect the mRNA expression of Cas9 and mPGES-1, and DNA was used for the T7E1 assay. As shown in Figure 1(C), PX459:sgRNA2 had superior results in Cas9 mRNA expression, mPGES-1 knockdown, and gene editing efficiency. It was therefore used in the subsequent experiment.

The mPGES-1 donor plasmid was constructed on the basis of pET32-2A-tdTomato-loxP-CAG-neo-loxP. After designing primers (Table 1), PCR was performed to obtain the sequences located upstream and downstream of the stop codon of the target gene mPGES-1, namely, left arm and right arm sequences (excluding the stop codon), which were recombined into pET32-2A-tdTomato. After sequencing, the sequence was verified before use. The 2A-tdTomato-loxP-CAG-neo-loxP sequence was located in the middle of the left and right arms. Liver-derived cells HepG2, HepG2.2.15, Huh7, BEL-7404, HL7702, and MHCC97H were selected, and the basic expression of mPGES-1 protein was detected via Western blot. The abundance of mPGES-1 protein expression was consistent with the construction of reporter cells in HL7702 and BEL-7404 cells (Figure 1(D)). The donor and PX459:sgRNA2 plasmids were cotransfected into HL7702 cells or BEL-7404 cells at a ratio of 3:1 and then screened by G418 for 2 weeks (Figure 1(E)). Afterward, monoclonal cells were selected via limited dilution in 96-well plates. After extensive culturing, numerous monoclonal cell lines expressing red fluorescent protein (Figure 1(F)) were obtained and used for further validation.

### Verification of mPGES-1 fluorescence reporter cells

**Figure 2.**
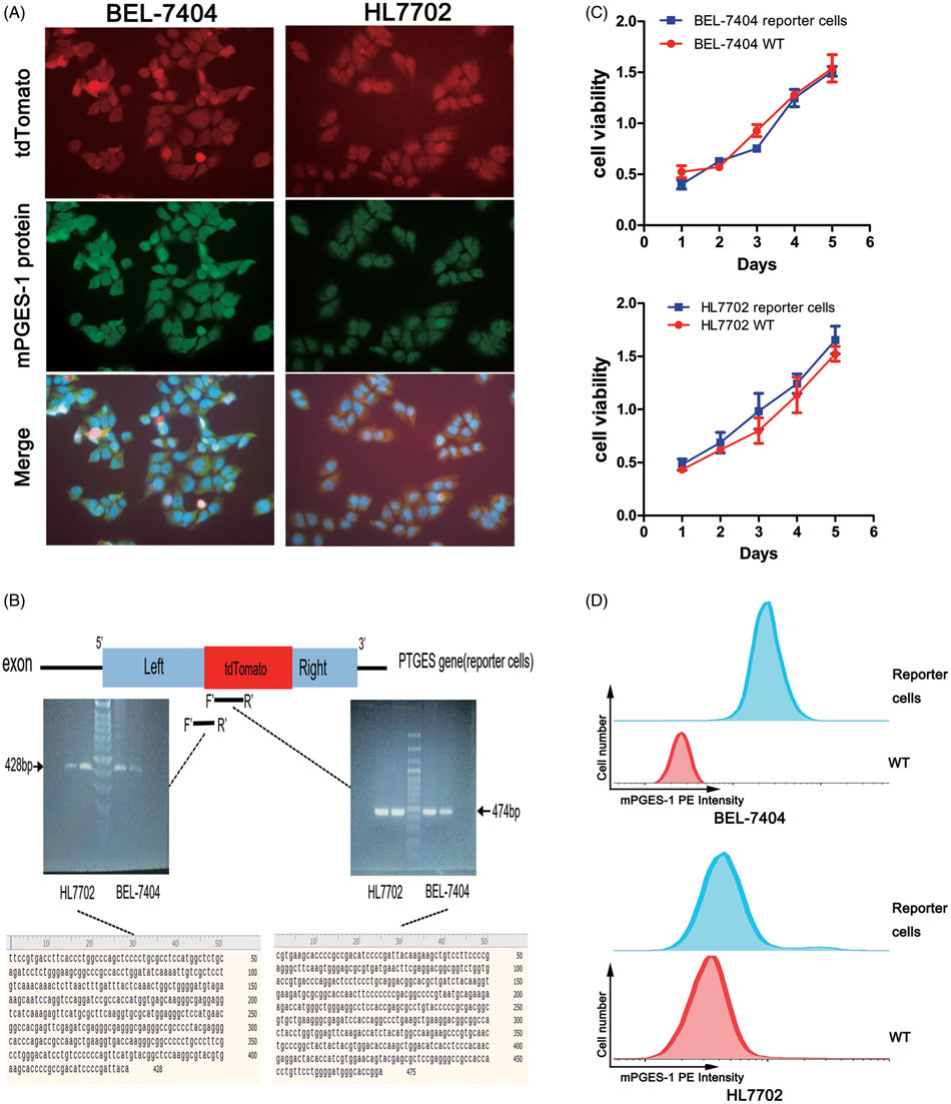
Verification of mPGES-1 fluorescence reporter cells. (A) Immunofluorescence staining was performed to verify the reporter cells. BEL-7404 and HL7702 reporter cells were cultured in six-well plates, and mPGES-1 primary antibody and FITC fluorescent secondary antibody were incubated to express green fluorescence. The cells expressed red fluorescence of tdTomato. After Merge + DAPI, the red-green fluorescence was superimposed to yellow, indicating that they were expressed in the same position. (B) Sanger sequencing verifies the accuracy of gene knock in in the reporter cells. Primers were designed in the tdTomato sequence and the left-tdTomato sequence, respectively, to extract the reporter cell DNA for PCR reaction, and the PCR product was used for electrophoresis gel imaging and sequencing. The gel imaging showed that the PCR band was within the expected product range. The sequencing results were also consistent with the expected results after blast. (C) CCK-8 assay was used to detect the viability of the reporter cells. The cells were inoculated into 96-well plates according to the cell density of 0.2 × 10^4^ per well. The cell viability was detected by spectrophotometry at 1 day, 2 days, 3 days, 4 days, and 5 days, respectively. OD450 nm–OD600 nm was the final value. (D) Detection of PE channel signals in reporter cells. The cells were inoculated into six-well plates and digested into a single cell after 48 h. The cells were washed with aseptic PBS for several times and filtered over 300 mesh. The signal intensity of the reporter cells and WT cells in PE channel was detected by flow cytometry.

To identify whether the monoclonal red fluorescent cell line is the mPGES-1 reporter cell expected in this experiment, we used immunofluorescence colocalisation and Sanger sequencing. In the immunofluorescence colocalisation experiment, we selected a fluorescent secondary antibody capable of expressing FITC. By binding to the mPGES-1 primary antibody, the target gene expressed green fluorescence under a fluorescence microscope. The fluorescent antibody-tagged mPGES-1 protein and the red fluorescent protein of the cell line were compared and found to be expressed in the same position in the cell (Figure 2(A)), thereby indicating that the gene editing target was correct.

According to the knock-in principle, the sequence of 2A-tdTomato was integrated into the genome of the cell via HDR. Therefore, we designed primers for the tdTomato and the left arm-tdTomato sequences (Table 1) and performed PCR using the template from the DNA of the red fluorescent cells. The PCR products were subjected to electrophoresis gel imaging and sequencing. As shown in Figure 2(B), the position of the bands in the gel imaging was as expected, and the sequencing results were accurate.

In summary, the double verification of immunofluorescence colocalisation and sequencing indicated that we successfully established mPGES-1 reporter cells. No difference was observed in cell viability between the constructed reporter cells and wild-type (WT) cells (Figure 2(C)), thereby indicating that the proliferation function of the engineered reporter cells was not affected. Moreover, FCM analysis showed that the PE intensity in reporter cells was remarkably enhanced compared with that of WT (Figure 2(D)). The intensity of fluorescence was detected via fluorescence microscopy, fluorescent microplate reading, and FCM, which were simple and convenient. Therefore, we predicted whether the expression of mPGES-1 was indirectly reflected by fluorescent signals, enabling reporter cells to be applied to drug screening.

### mPGES-1 expression in reporter cells by IL-1β stimulation and mPGES-1-siRNA treatment

**Figure 3.**
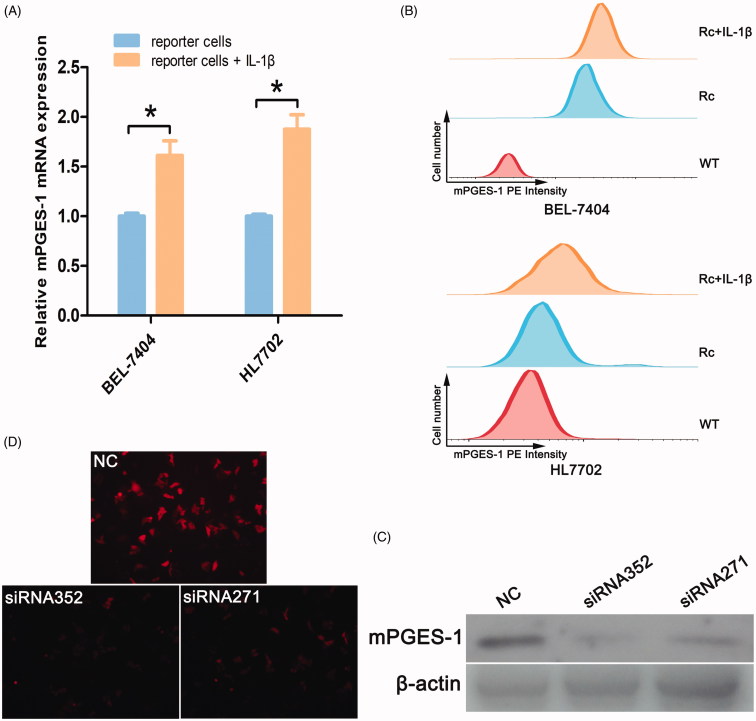
mPGES-1 expression in reporter cells by IL-1β stimulation and mPGES-1-siRNA treatment. (A) Expression of mPGES-1 mRNA in reporter cells stimulated by IL-1β. The reporter cells were seeded in six-well plates, including the experimental group with IL-1β stimulation (2.5 ng/ml) for 24 h and the control group. RNA was extracted until the time of full growth, and the expression of mPGES-1 mRNA was detected by real-time fluorescent quantitative PCR. And *p< .05, *n* = 3. (B) The expression of red fluorescent signal was detected by FCM after the cells were stimulated by IL-1β. The blank control group (WT), the negative control group (Rc, reporter cells) and the experimental group (Rc + IL-1β) were designed. The cells were seeded in a six-well plate, cultured and treated according to the experimental design. The digested cells were blown into single cells, washed with PBS, filtered through 300 mesh, and the signal of the PE channel was detected by flow cytometry. (C) Checking the knockout effect of mPGES-1 siRNA. Two pairs of siRNA and NC were transiently transfected into BEL-7404 wild-type cells. After 48 h, the protein was extracted, and 30 μg was used to detect the knock down of mPGES-1 protein by Western blot. (D) Fluorescence intensity changes of reporter cells after mPGES-1 siRNA treatment. NC, siRNA352, and siRNA271 were transiently transfected into BEL-7404 reporter cells, respectively, and the difference in red fluorescence intensity was observed 72 h after transfection. The fluorescence intensity of the reporter cells transfected with siRNA was significantly lower than that of the NC group.

Human mPGES-1 is encoded by the *PTGES* (prostaglandin E synthase) gene and can be induced by the proinflammatory cytokine IL-1β. After treatment with IL-1β (2.5 ng/mL), the expression level of mPGES-1 mRNA increased (Figure 3(A)), and FCM results showed that the PE intensity was enhanced (Figure 3(B)). Two pairs of siRNAs (siRNA352 and siRNA271) were designed for the *PTGES* gene. siRNA was transfected into BEL-7404 WT cells, and protein was extracted 48 h after transfection. Western blot indicated that siRNA352 and siRNA271 had the *PTGES* knockdown effect (Figure 3(C)), but the effect of siRNA352 (knockdown by 74%) was more effective than that of siRNA271. Two pairs of siRNAs were transiently transfected into reporter cells. After 72 h, the expression of red fluorescent protein was observed via fluorescence microscopy. The red fluorescence was found to be considerably attenuated in the reporter cells transfected with siRNA compared with normal reporter cells (Figure 3(D)). The enhancement of fluorescence intensity by IL-1β and the inhibitory effect of siRNA also fully confirmed the accurate insertion of the fluorescent tag.

### mPGES-1 fluorescence reporter cells can be applied to the screening of small molecule drug functions

**Figure 4.**
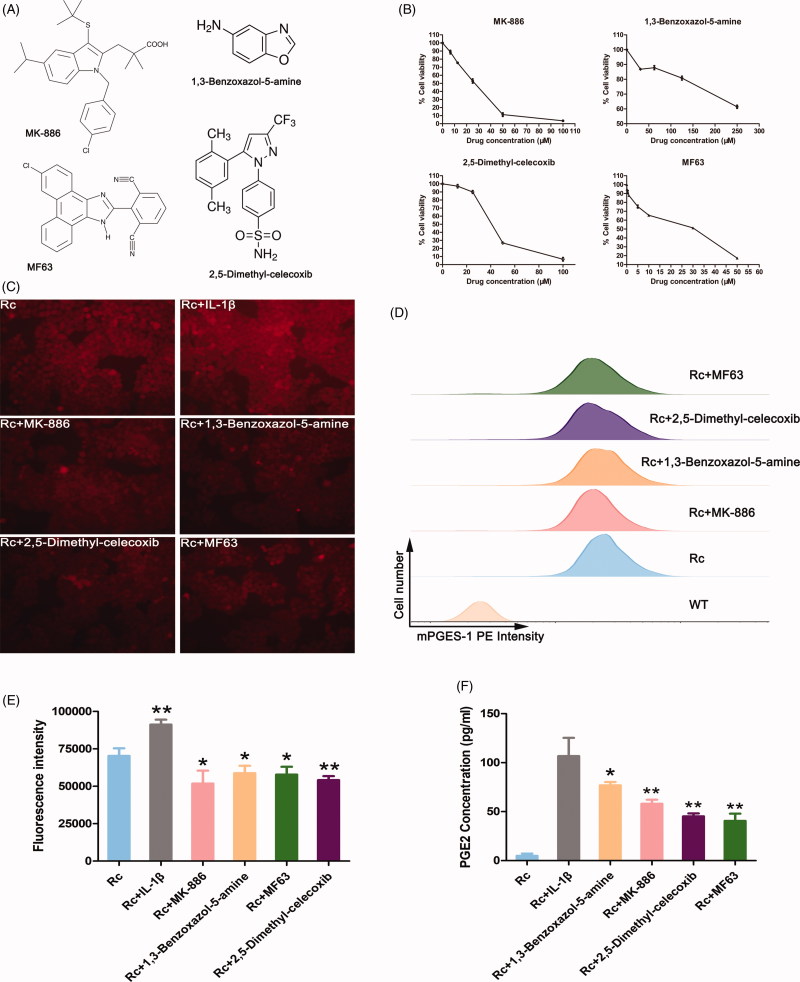
mPGES-1 fluorescence reporter cells can be applied to the screening of small molecule drug functions. (A) Structure diagram of four mPGES-1 inhibitors. (B) The dose–response curve of mPGES-1 inhibitors. Cell viability was assessed using the CCK-8 assay. (C) Changes in red fluorescence intensity of BEL-7404 reporter cells treated with inhibitors and IL-1β. After the cells were treated with the inhibitor IC20 concentration for 72 h, the expression of red fluorescence intensity was observed under a fluorescence microscope. The experimental group added with IL-1β only needed to stimulate for 24 h. (D) Changes in PE intensity of BEL-7404 reporter cells after treatment with inhibitors. BEL-7404 reporter cells were seeded in six-well plates and treated with IC20 concentrations of the four inhibitors for 72 h. The cells were treated with flow cytometry to detect PE intensity, and PE intensity from WT cells were used as blank controls. Rc (reporter cells) as a negative control. (E) The expression of tdTomato fluorescence intensity in BEL-7404 reporter cells after treatment with inhibitors and IL-1β. BEL-7404 reporter cells were seeded in black 96-well plates with opaque walls at a cell density of 0.5 × 10^4^ per well. After 24 h, IL-1β (stimulation for 24 h) and four different small molecule inhibitors (treated for 72 h) were added. The 96-well plate was placed in a fluorescent microplate reader to measure the fluorescence intensity. The excitation light wavelength was 554 nm, the emission light wavelength was 581 nm, and Rc was a negative control group (*p< .05, **p< .005, *n* = 5). (F) The level of PGE2 concentration in BEL-7404 reporter cells after treatment with inhibitors and IL-1β. Reporter cells were seeded in six-well plates at a cell density of 4 × 10^5^ per well. “Rc + IL-1β”: reporter cells + IL-1β (stimulation for 24 h). And “Rc + inhibitors”: reporter cells + molecule inhibitors (treated for 72 h)+IL-1β (stimulation for 24 h). The secretion level of PGE2 was detected by ELISA according to the manufacturer's instructions. And “Rc + IL-1β” as a control group (*p< .05, **p< .005, *n* = 4).

To identify whether reporter cells were suitable for screening small molecule drugs, four mPGES-1 inhibitors, namely, MK-886, 1,3-benzoxazol-5-amine, 2,5-dimethyl-celecoxib and MF63 (Figure 4(A)), were selected to treat BEL-7404 reporter cells. To reduce the potential bias introduced by the variation in growth inhibition caused by small molecule inhibitors, we established a dose–response curve for each small molecule inhibitor in BEL-7404 cells (Figure 4(B)) and used inhibitory concentration 20% (IC20) of the inhibitor. After the cells were treated with IC20 concentrations of the inhibitors for 72 h, the red fluorescence of the reporter cells decreased with fluorescence microscopy (Figure 4(C)). To avoid subjective influence in fluorescence microscopy, we used FCM and a fluorescence microplate reader to monitor the change of fluorescence value semiquantitatively. The PE intensity of the reporter cells after FCM detection weakened in varying degrees (Figure 4(D)). The fluorescence intensity also weakened in the fluorescence microplate reader (Figure 4(E)), indicating that the small molecule drugs exerted mPGES-1 inhibition. In addition, the level of PGE2 after inhibitor treatment was detected to indirectly reflect the effect of drugs on mPGES-1 enzyme activity. The results (Figure 4(F)) showed that the level of PGE2 secretion in the supernatant decreased in varying degrees after the reporter cells were treated with different inhibitors. Therefore, the constructed reporter cells were suitable for the screening of small molecule drugs and were combined with FCM or fluorescent microplate reader to establish a platform for the screening of large quantities of mPGES-1 inhibitors.

## Discussion

In this study, an exogenous sequence (tdTomato tag) was introduced into the liver cell genome via the CRISPR/Cas9 technology, and mPGES-1 reporter cells were established. tdTomato is bright and stable. The reporter cells that obtained the tag can monitor the expression of the mPGES-1 gene via cell imaging and can be applied to the screening of mPGES-1 inhibitors.

Based on the principle of the CRISPR/Cas9 system knock-in and the HDR pathway, we inserted the tdTomato gene into the stop codon position of the chromosomal mPGES-1 gene. The expression of both genes was under the transcriptional regulation of the mPGES-1 promoter. The fluorescence intensity of tdTomato was positively correlated with the expression of mPGES-1. Factors that possibly affected donor integration efficiency were sgRNA cleavage efficiency and microhomology between the donor vector and the genome^20–22^. Therefore, to improve integration efficiency, we rigorously screened and selected sgRNAs with better editing effects and used homologous arms with a total homology of more than 2.5 kb in the donor vector design. This experiment was also rigorous in the validation and application of reporter cell lines, including the following. First, the reporter cells carrying tdTomato were enhanced more in PE channel signals than in WT cells. Second, the fluorescence intensity of the reporter cells treated with mPGES-1 siRNA was considerably reduced, and the PE intensity of IL-1β-stimulated mPGES-1 reporter cells was enhanced. Furthermore, in the immunofluorescence colocalisation, the green fluorescently labelled mPGES-1 protein and the red fluorescent protein of the reporter cells were localised in the same position in the cell, thereby indicating that the gene editing target was accurate. Sanger sequencing results showed that the tdTomato sequence was successfully integrated into the endogenous mPGES-1 gene and was stably expressed. Finally, the successful construction of reporter cells played an important role in the screening of small molecule inhibitors with the advantages of their fluorescence imaging.

In our experimental design, when the Cas9 protein exerted a cleavage function to form DSB, a HDR vector was introduced, thereby achieving the integration of the foreign gene. In constructing reporter cells, the HDR is more precise and feasible than the NHEJ model. The homology template is highly specific for the target gene. However, a specific homologous template must still be designed and synthesised for the different target genes to be labeled^23^, which is sometimes expensive and laborious.

In current CRISPR technology research, some scholars have discovered substantial fragment deletions or other fragment deletions, chromosomal translocations, and other complex DNA recombination problems near the Cas9 cleavage site^24^^,^^25^, particularly the deletion of the tumour suppressor gene p53^26^^,^^27^. We were concerned that cell proliferation may be impaired after using gene editing techniques, thereby leading to false results in the use of reporter cells for small molecule drug screening. To detect the presence or absence of this possibility, we tested the cell proliferation ability of the reporter cells and found that cell proliferation in the reporter and WT cells were the same (statistically meaningless). Thus, the possibility of editing techniques affecting cell proliferation was initially ruled out.

mPGES-1 is a terminal enzyme for the synthesis of PGE2, and targeting inhibition of mPGES-1 does not cause changes in other prostaglandins, and thus, has research significance. At present, research on mPGES-1 is increasing, and it has been reported that the inhibition of targeting mPGES-1 has many effects: promoting liver repair after injury in mice^28^, inhibiting the progression and occurrence of experimental cholangiocarcinoma^29^, inhibiting the growth of neuroblastoma^30^^,^^31^, and enhancing antiviral immunity^32^. Qualified mPGES-1 inhibitors can circumvent the serious side effects of COX-2 inhibitors^8^^,^^9^, and targeted inhibition of mPGES-1 will play an important role in anti-inflammatory and tumour treatment^33^^,^^34^.

In summary, we used the CRISPR/Cas9 technology to create reporter cells and monitor the expression of endogenous mPGES-1 via fluorescence imaging visualisation. Moreover, the commonly used mPGES-1 inhibitor treatment experiments also demonstrated that the reporter cell line is suitable for drug screening. By binding mPGES-1 reporter cells to high-throughput imaging systems, the extensive screening of mPGES-1 inhibitors will be convenient, efficient, and inexpensive.
